# AntiCD3Fv fused to human interleukin-3 deletion variant redirected T cells against human acute myeloid leukemic stem cells

**DOI:** 10.1186/s13045-015-0109-5

**Published:** 2015-02-28

**Authors:** Dongmei Fan, Zhenzhen Li, Xiaolong Zhang, Yuqi Yang, Xiangfei Yuan, Xiuli Zhang, Ming Yang, Yizhi Zhang, Dongsheng Xiong

**Affiliations:** State Key Laboratory of Experimental Hematology, Institute of Hematology & Hospital of Blood Diseases, Chinese Academy of Medical Sciences and Peking Union Medical College, Tianjin, 300020 People’s Republic of China; School of Pharmacy, Tianjin Medical University, Tianjin, 300070 People’s Republic of China; Central Hospital of Karamay, Karamay, Xinjiang 834000 China

**Keywords:** AntiCD3Fv, CD123, Fusion proteins, Leukemic stem cells, T cell

## Abstract

**Background:**

Leukemic stem cells (LSCs) are frequently seen as a cause of treatment failure and relapse in patients with acute myeloid leukemia (AML). Thus, successful new therapeutic strategies for the treatment of AML should aim at eradicating LSCs. The identification of targets on the cell surface of LSCs is getting more and more attention. Among these, CD123, also known as the interleukin-3 (IL3)-receptor α chain, has been identified as a potential immunotherapeutic target due to its overexpression on LSCs in AML as well as on AML blasts, rather than normal hematopoietic stem cells.

**Methods:**

We constructed a CD123-targeted fusion protein antiCD3Fv-⊿IL3, with one binding site for T cell antigen receptor (TCRCD3) and the other for CD123, by recombinant gene-engineering technology. Cysteine residues were introduced into the V domains of the antiCD3Fv segment to enhance its stability by locking the two chains of Fv together with disulfide covalent bonds. The stability and cytotoxicity of the two fusion proteins were detected *in vitro* and *in vivo*.

**Results:**

Both fusion proteins were produced and purified from *Escherichia coli* 16C9 cells with excellent yields in fully active forms. High-binding capability was observed between these two fusion proteins and human IL3R, leading to the specific lysis of CD123-expressing cell lines KG1a; also, mononuclear cells from primary AML patients were inhibited in a colony forming assay *in vitro*, presumably by redirecting T lymphocytes *in vitro*. In addition, they displayed an antileukemic activity against KG1a xenografts in non-obese diabetic/severe combined immunodeficient (NOD/SCID) mice, especially disulfide-stabilized (ds)-antiCD3Fv-⊿IL3 for its improved stability.

**Conclusions:**

These results suggest that both fusion proteins display the antileukemic activity against CD123-expressing cell lines as well as leukemic progenitors *in vitro* and *in vivo*, especially ds-antiCD3Fv-⊿IL3. They could be the promising candidates for future immunotherapy of AML.

**Electronic supplementary material:**

The online version of this article (doi:10.1186/s13045-015-0109-5) contains supplementary material, which is available to authorized users.

## Background

Acute myeloid leukemia (AML) is characterized as the rapid proliferation of immature myeloid cells in the bone marrow, resulting in dysfunctional hematopoiesis [1]. Although most patients can achieve an initial complete remission with standard induction chemotherapy, the majority of them eventually relapse and become resistant to cytotoxic chemotherapy [2]. Leukemic stem cells (LSCs) are a common cause of the treatment failure and the relapse in these patients [3-5], and moreover, their gene signature are significantly associated with the prognosis of human AML [6]. Therefore, development of novel therapeutics that can selectively target LSCs is crucial. Recently, many researchers have identified several AML-associated cell surface phenotypic markers that can be used as future therapeutic targets [7]. Among them, CD123, also known as the interleukin-3 (IL3) receptor α chain, has received a considerable amount of interest, due to its overexpression in LSCs in AML rather than normal hematopoietic stem cells [7,8]. Pre-clinical studies using a monoclonal antibody targeting CD123 (7G3) or diphtheria toxin fused to interleukin-3 (DT388IL3) for the treatment of AML have demonstrated promising antileukemic activity [9-11].

A more potentially efficient approach for cancer prevention and treatment is to manipulate the immune system of patients toward cancers cells [12,13]. As we know, T cells play a pivotal role in tumor immunity by directly eliminating tumor cells through formation of synapses of cytotoxic T cell tumor cells [14]. However, T cells usually cannot effectively identify tumor cells or become anergic because of the complexity of the T cell recognition mechanism [15]. In the past two decades, scientists have already taken advantage of T cell potency in cancer therapy by redirecting their specificity toward cell surface tumor-associated antigens. For example, bispecific antibodies, by simultaneously recognizing target antigen on the surface of cancer cells and an activating receptor on the surface of an immune effector cell, offer an opportunity to redirect immune effector cells to kill cancer cells. The other approach is the generation of chimeric antigen receptors (CAR) by fusing extracellular antibodies to intracellular-signaling domains. And then, the CAR-engineered T cells are able to specifically kill tumor cells in a major histocompatibility complex (MHC)-independent way. The above two approaches, both showing promising clinical results, are recognized as the most effective immuno-biological therapeutics for tumors [16]. Recently, Kuo et al. described a bispecific single-chain Fv (scFv) immunofusion or BIf to target CD123+ leukemia *in vitro* that contains an antiCD123 scFv fused at the N-terminus of human IgG1 hinge-C_H_2-C_H_3 and an antiCD3 scFv fused at C-terminus [17]. While, Mardiros et al. developed two CD123 CAR-redirected T cells mediated potent effector activities against CD123+ cell lines as well as primary AML patient samples *in vitro* and *in vivo* [18]. Similarly, Sarah Tettamanti et al. have constructed CD123-specific CARs that can strongly enhance antiAML CIK functions [19]. All these works proved the effectiveness of the CD123-retargeted T cell therapy.

IL3 is a cytokine that promotes the proliferation and differentiation of multipotential and committed myeloid and lymphoid progenitors [20]. The IL3 receptor is a heterodimeric structure composed of α and β subunits. The α chain (CD123) directly binds IL3, and the β subunit is used to conduct signals [21]. The ligand-receptor-binding activity is considered to be very potent. To further increase the stability of the ligand-receptor binding, combinatorial mutagenesis studies by several laboratories showed that deletion of eight C-terminal amino acid residues from IL3 (⊿125-133) or the variant K116W resulted in even higher affinity interactions with IL3R and greater cytotoxicity against human leukemic stem cells [22-25].

Based on these previous findings, here we constructed a similar fusion protein antiCD3Fv-⊿IL3 (with the C-terminal eight amino acids of IL3 deleted, ⊿125-133), just as bispecific antibodies, that is theoretically capable of recruiting a polyclonal T cell against LSCs that express CD123, with one of its arms to the common T cell signaling protein CD3 and the other to the tumor-associated antigen CD123 on the target LSCs. Moreover, to enhance the stability of the fusion protein, a disulfide-stabilized format (ds-antiCD3Fv-⊿IL3) of this fusion protein was generated by locking the two chains of Fv together with disulfide covalent bonds. High-binding capability was observed between these two fusion proteins and human IL3R, leading to the specific lysis of CD123-expressing cell lines KG1a; also, mononuclear cells from primary AML patients were inhibited in a colony-forming assay *in vitro*, presumably by redirecting T lymphocytes *in vitro. In vivo*, they displayed an antileukemic activity against KG1a xenografts in non-obese diabetic/severe combined immunodeficient (NOD/SCID) mice. And the ds-antiCD3Fv-⊿IL3 demonstrated an even better performance due to its higher stability. Two advantages make the fusion proteins more effective: first, they were composed of antiCD3Fv and human recombinant IL3, resulting in low immunogenicity; second, ⊿IL3, the ligand of CD123, was employed, demonstrating a better binding activity than the antigen-antibody binding.

## Results

### Construction and expression of fusion proteins antiCD3Fv-⊿IL3 and ds-antiCD3Fv-⊿IL3

**Figure 1:**
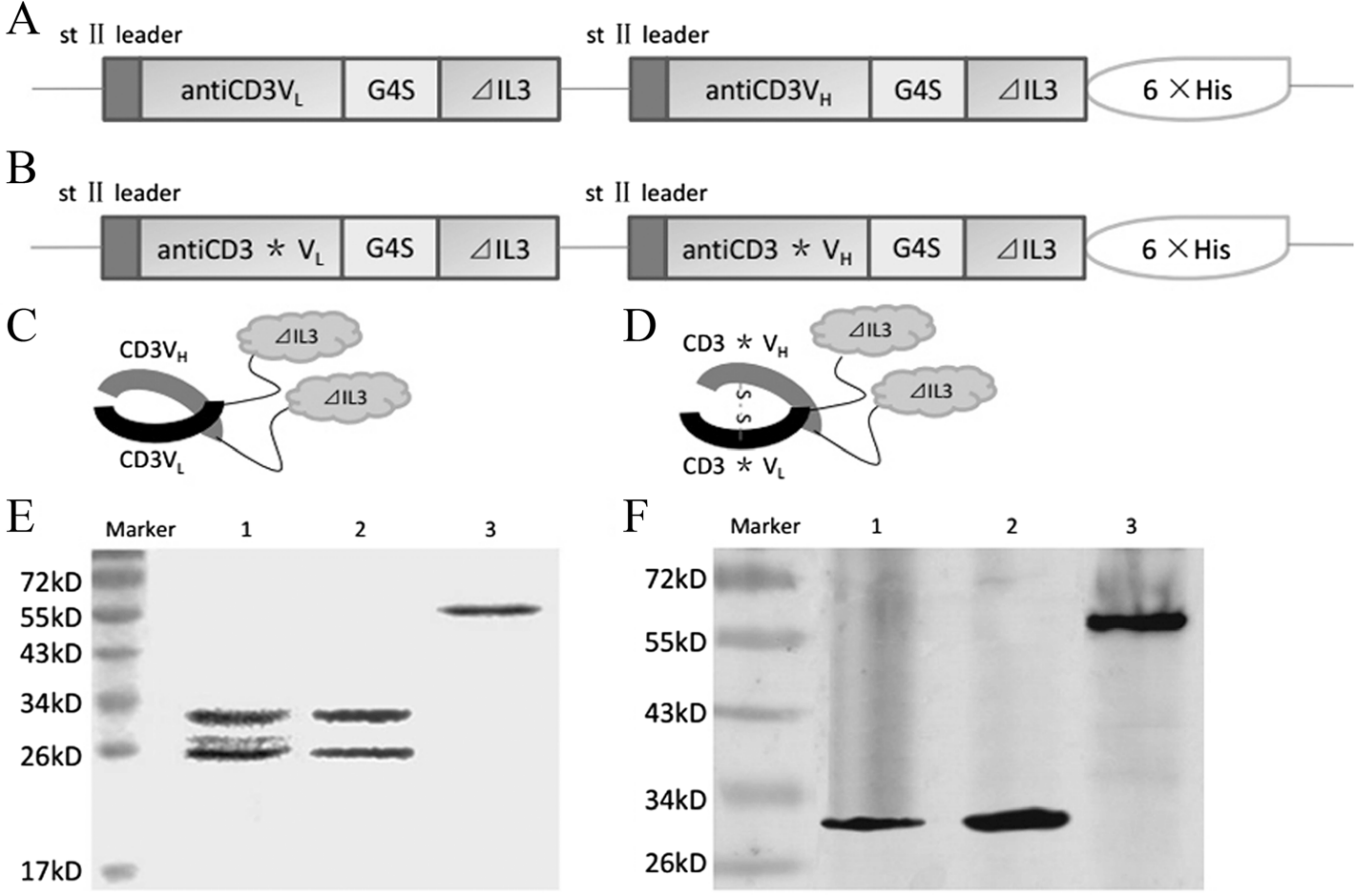
**Expression and purification of the fusion proteins antiCD3Fv-**⊿**IL3 and the ds-antiCD3Fv-**⊿**IL3.** Schematic of the expression plasmid for **(A)** antiCD3Fv-⊿IL3 and **(B)** ds-antiCD3Fv-⊿IL3, and structure of the fusion proteins for **(C)** antiCD3Fv-⊿IL3 and **(D)** ds-antiCD3Fv-⊿IL3. Note: the drawing is not to scale; asterisk (*) indicates the site of the disulfide bond. The fusion proteins were expressed in *E. coli*, purified by affinity chromatography, and analyzed by SDS-PAGE and immunoblotting. **(E)** Colloidal staining of a SDS-PAGE gel. Lane 1: antiCD3Fv-⊿IL3 in non-reducing loading buffer; lane 2: ds-antiCD3Fv-⊿IL3 in reducing loading buffer; lane 3: ds-antiCD3Fv-⊿IL3 in non-reducing loading buffer. **(F)** Immunoblot analysis of the SDS-PAGE gel with an anti-His-tag antibody. Lane 1: antiCD3Fv-⊿IL3 in non-reducing loading buffer; lane 2: ds-antiCD3Fv-⊿IL3 in reducing loading buffer; lane 3: ds-antiCD3Fv-⊿IL3 in non-reducing loading buffer.

The nucleotide sequences encoding the fusion proteins were verified by DNA sequencing. The last eight amino acids at the C-terminal of IL3 were entirely deleted. In addition, the Ser-100 of antiCD3V_L_ and Gly-44 of antiCD3V_H_ were successfully replaced by cysteines (Figure 1B). The fusion proteins were expressed by cosecretion of the two polypeptide chains (antiCD3V_L_-⊿IL3 or antiCD3*V_L_-⊿IL3 and antiCD3V_H_-⊿IL3-His or antiCD3*V_H_-⊿IL3-His) from *Escherichia coli* 16C9 cells as periplasmic native proteins (Figure 1A,B). Then, antiCD3V_L_-⊿IL3 and antiCD3V_H_-⊿IL3-His were folded to form fusion protein antiCD3Fv-⊿IL3 depending on the intermolecular force (Figure 1C) whereas the two cysteine-mutated polypeptide chains antiCD3*V_L_-⊿IL3 and antiCD3*V_H_-⊿IL3-His formed fusion protein ds-antiCD3Fv-⊿IL3 relying on the disulfide bonds in the periplasmic space (Figure 1D). The fusion proteins were released from the periplasmic space of *E. coli* by osmotic shock and purified by 6 × His-tag affinity chromatography. The yields of purified fusion proteins ranged from 1 to 2 mg/L of culture medium.

The purified fusion proteins were analyzed by SDS-PAGE and Western blot. The fusion protein antiCD3Fv-⊿IL3 was resolved under electrophoretic conditions and detected as two bands of approximately 30 and 27 kD, corresponding to the two polypeptide chains of antiCD3V_H_-⊿IL3 and antiCD3V_L_-⊿IL3, respectively, as anticipated (Figure 1E). Under reducing conditions, the fusion protein ds-antiCD3Fv-⊿IL3 was resolved into two proteins bands consistent with those of antiCD3Fv-⊿IL3. However, under non-reducing conditions, the fusion protein ds-antiCD3Fv-⊿IL3 was detected as one band at approximately 57 kD (Figure 1E). Western blot analysis using an anti-His-tag antibody validated the existence of the His-tag-containing fragment and confirmed that the two polypeptide chains of antiCD3*V_L_-⊿IL3 and antiCD3*V_H_-⊿IL3 were linked by the disulfide bond (Figure 1F).

### Dual specificity of the fusion proteins antiCD3Fv-⊿IL3 and ds-antiCD3Fv-⊿IL3

**Figure 2:**
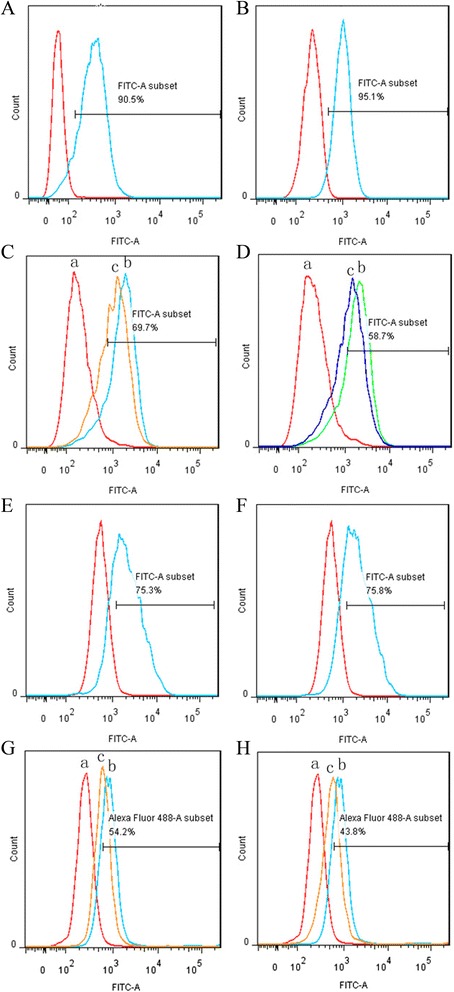
**Analysis of specific binding of fusion proteins antiCD3Fv-**⊿**IL3 and ds-antiCD3Fv-**⊿**IL3 to Jurkat and KG1a, as well as the competitive binding activity with monoclonal antibody HIT3a or CD123. (A, B)** Jurkat cells were incubated with fusion proteins **(A)** antiCD3Fv-⊿IL3 or **(B)** ds-antiCD3Fv-⊿IL3. **(E-F)** KG1a cells were incubated with **(E)** antiCD3Fv-⊿IL3 or **(F)** ds-antiCD3Fv-⊿IL3. Note: the gate represented the positive rate of binding of fusion proteins. In the competitive binding assay, **(C, D)** Jurkat cells were first incubated with (**(C)**: c) antiCD3Fv-⊿IL3 or (**(D)**: c) ds-antiCD3Fv-⊿IL3 and HIT3a. **(G, H)** KG1a cells were first incubated with (**(G)**: c) antiCD3Fv-⊿IL3 or (**(H)**: c) ds-antiCD3Fv-⊿IL3 and mAb CD123, then incubated with an FITC-conjugated antimouse IgG. Control groups only reacted with (a.) PBS and (b.) parent mAb IgG. Note: the gate represented the binding rate of the line of c with monoclonal antibody HIT3a or CD123.

Both antiCD3Fv-⊿IL3 and ds-antiCD3Fv-⊿IL3 bind to CD123-positive KG1a cells and CD3-positive Jurkat cells with similar efficiency as their parental monoclonal antibodies CD123 and HIT3a to the respective target cells (Figure 2A,B,E,F). Furthermore, both fusion proteins were able to partly reduce the binding efficiency of the parental monoclonal antibodies HIT3a and CD123 McAb to Jurkat and KG1a, respectively, in a competitive binding assay (Figure 2C,D,G,H). No significant decrease in binding activity of ds-antiCD3Fv-⊿IL3 fusion protein was detected compared to the parental fusion protein antiCD3Fv-⊿IL3.

### Stability of fusion proteins antiCD3Fv-⊿IL3 and ds-antiCD3Fv-⊿IL3 *in vitro*

**Figure 3:**
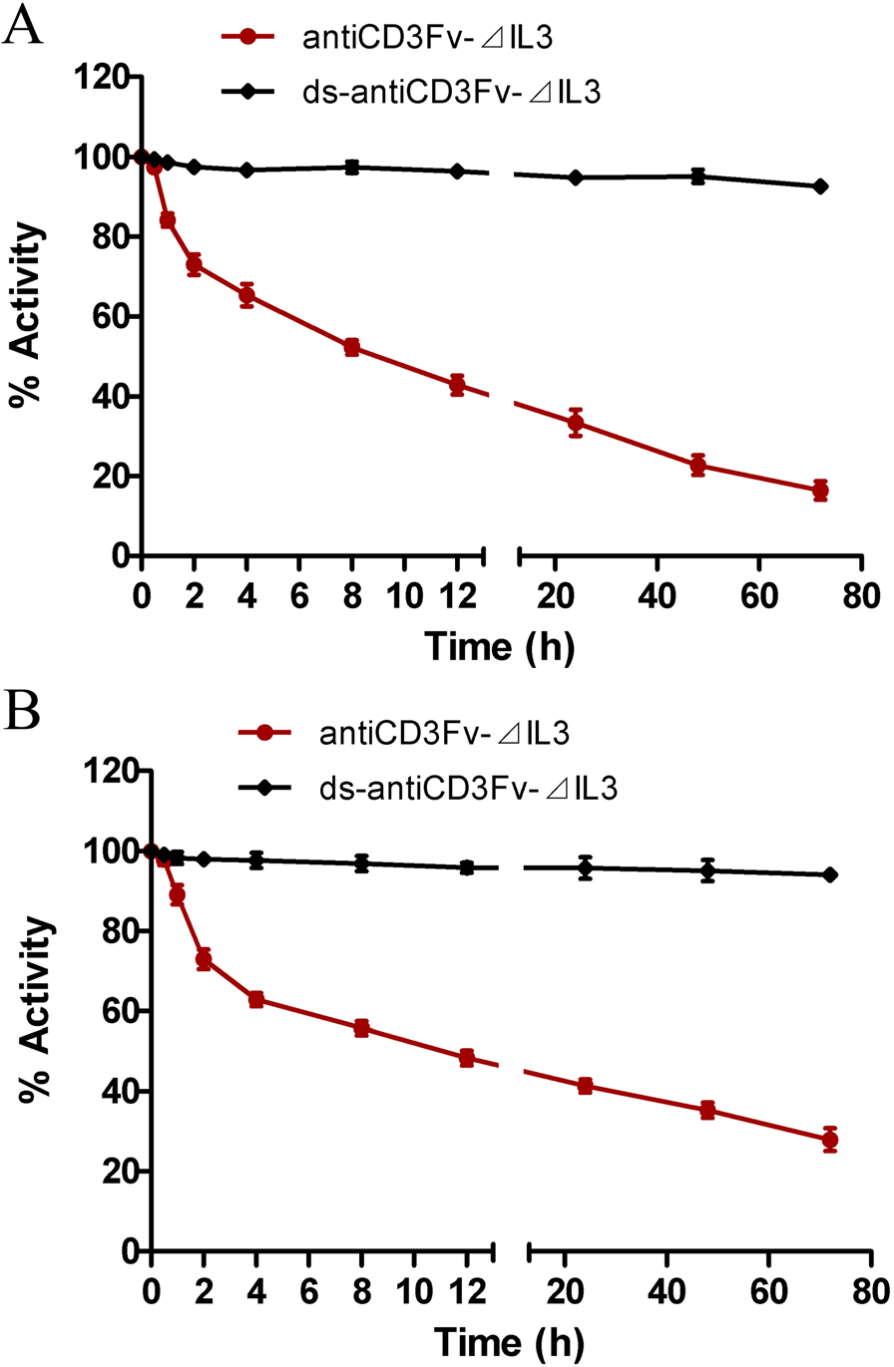
**Comparison of the stability of fusion proteins antiCD3Fv-**⊿**IL3 and ds-antiCD3Fv-**⊿**IL3** ***in vitro*** **.** The stability was determined by testing binding activity of fusion proteins to **(A)** CD3-positive Jurkat cells or **(B)** CD123-positive KG1a cells after incubation in 0.2% HSA at 37°C for a prolonged time period. Data were normalized to *t*_0_ (initial time), which was set at 100%. The data shown represent the averages of three independent experiments.

The stability of the fusion proteins *in vitro* was determined by analysis of their binding to target cells after incubation in PBS containing 0.2% (*w*/*v*) human serum albumin (HSA) at 37°C for several hours. The results showed that the binding capacity of the fusion proteins antiCD3Fv-⊿IL3 was reduced by nearly 50% after incubation for 6 h and 80% for 72 h, while the binding capacity of the ds-antiCD3Fv-⊿IL3 remained more than 90% after 72 h (Figure 3A,B).

### Tumor cell lysis mediated by fusion proteins antiCD3Fv-⊿IL3 and ds-antiCD3Fv-⊿IL3

**Figure 4:**
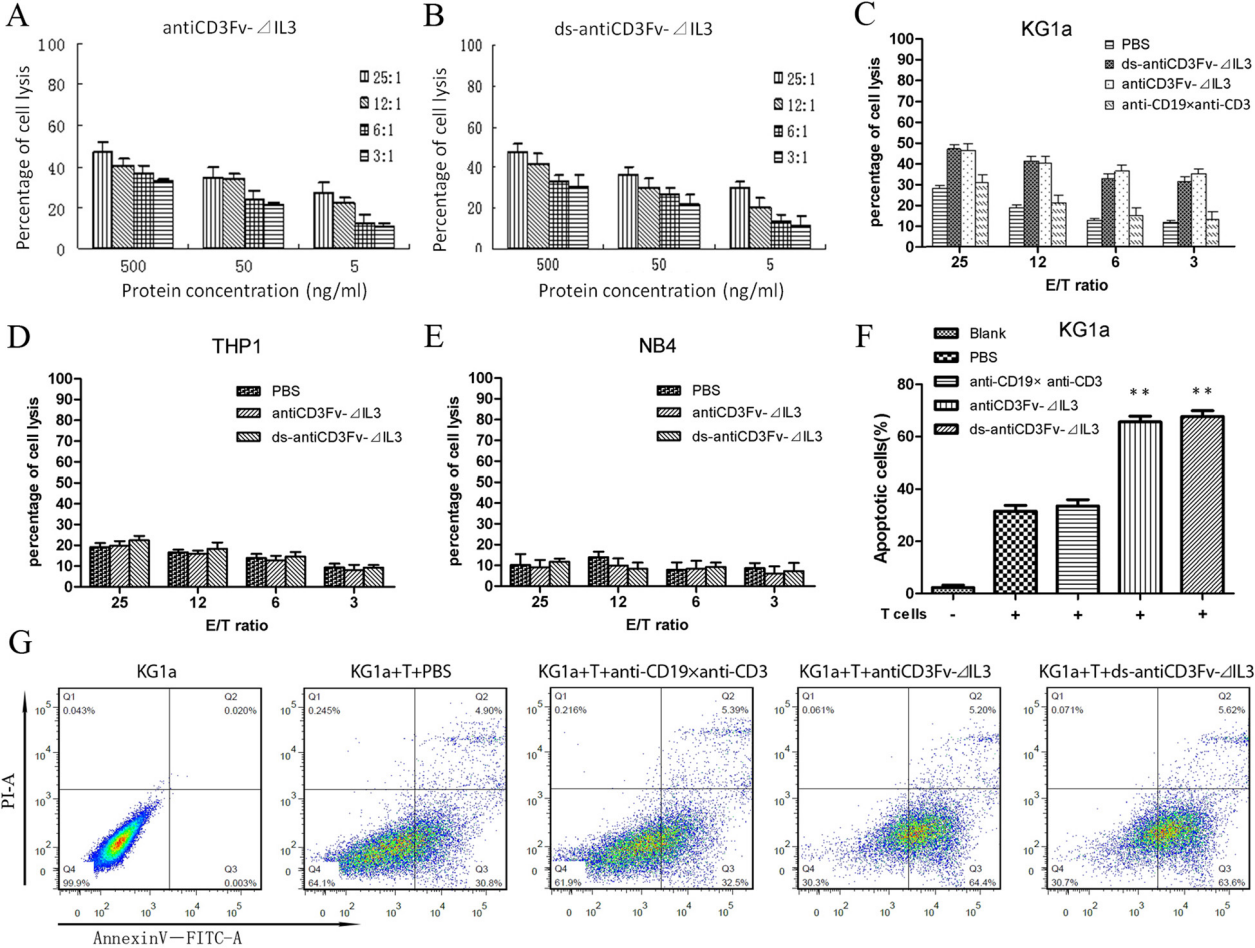
**Cytotoxicity of IL2 pre-activated human T cells to KG1a cells in different effector to target (E/T) ratios mediated by different concentrations of fusion proteins in a non-radioactive cytotoxicity assay. (A)** Cytotoxicity of T cells in the presence of antiCD3Fv-⊿IL3. **(B)** Cytotoxicity of T cells in the presence of ds-antiCD3Fv-⊿IL3. Concentrations of fusion proteins were different (500, 50, 5 ng/mL). E/T cell ratios ranged from 25:1 to 3:1. **(C)** Lysis of target cells by T cells mediated by PBS, ds-antiCD3Fv-⊿IL3, antiCD3Fv-⊿IL3, and the control diabody antiCD19 × antiCD3. The concentration of all fusion proteins was 500 ng/mL. CD123-negative cell line **(D)** THP1 and **(E)** NB4 were included as a control. **(F, G)** Ratios of apoptotic cells mediated by fusion proteins (500 ng/mL) combined with pre-activated T cells at E/T ratio of 25:1. PBS and antiCD19 × antiCD3 are used as a control. **P < 0.01 vs PBS. Data shown are the mean ± SD of three repeated experiments.

A non-radioactive cytotoxicity assay was performed to determine the ability of the fusion proteins antiCD3Fv-⊿IL3 and ds-antiCD3Fv-⊿IL3 to induce lysis of CD123+ tumor cells in the presence of pre-activated human T cells. The fusion proteins antiCD3Fv-⊿IL3 and ds-antiCD3Fv-⊿IL3 appeared to be potent in retargeting T cell lysis of the CD123-positive KG1a cells (Figure 4A,B,C), whereas CD123-negative cells THP1 and NB4 were not lysed under the same conditions (Figure 4D,E). Correspondingly, the ratio of apoptotic cells in the group of antiCD3Fv-⊿IL3 (65.633% ± 3.807%) and ds-antiCD3Fv-⊿IL3 (67.733% ± 3.821%) were increased when combined with pre-activated human T cells (Figure 4F,G). Additional movie files show this process of target cell lysis in more detail (see Additional file 1: Movie 1, Additional file 2: Movie 2, Additional file 3: Figure S1, and Additional file 4: Figure S2). Lysis of target cells mediated by the fusion protein or ds fusion protein increased in a dose-dependent manner: increasing either the concentration of fusion proteins or the ratio of effector to target cells resulted in enhanced target cell cytotoxicity (Figure 4A,B). The cytotoxic activity was not enhanced when an equal amount of the antiCD3/antiCD19 diabody was added, because CD19 was not expressed on the KG1a cells (Figure 4C). There was no statistical difference between the parental fusion protein and the ds fusion protein (500 ng/mL) in mediating the lysis of KG1a cells at various E:T ratios (Figure 4C).

### Cytotoxicity of T cells mediated by the fusion proteins against AML leukemic progenitors

**Figure 5:**
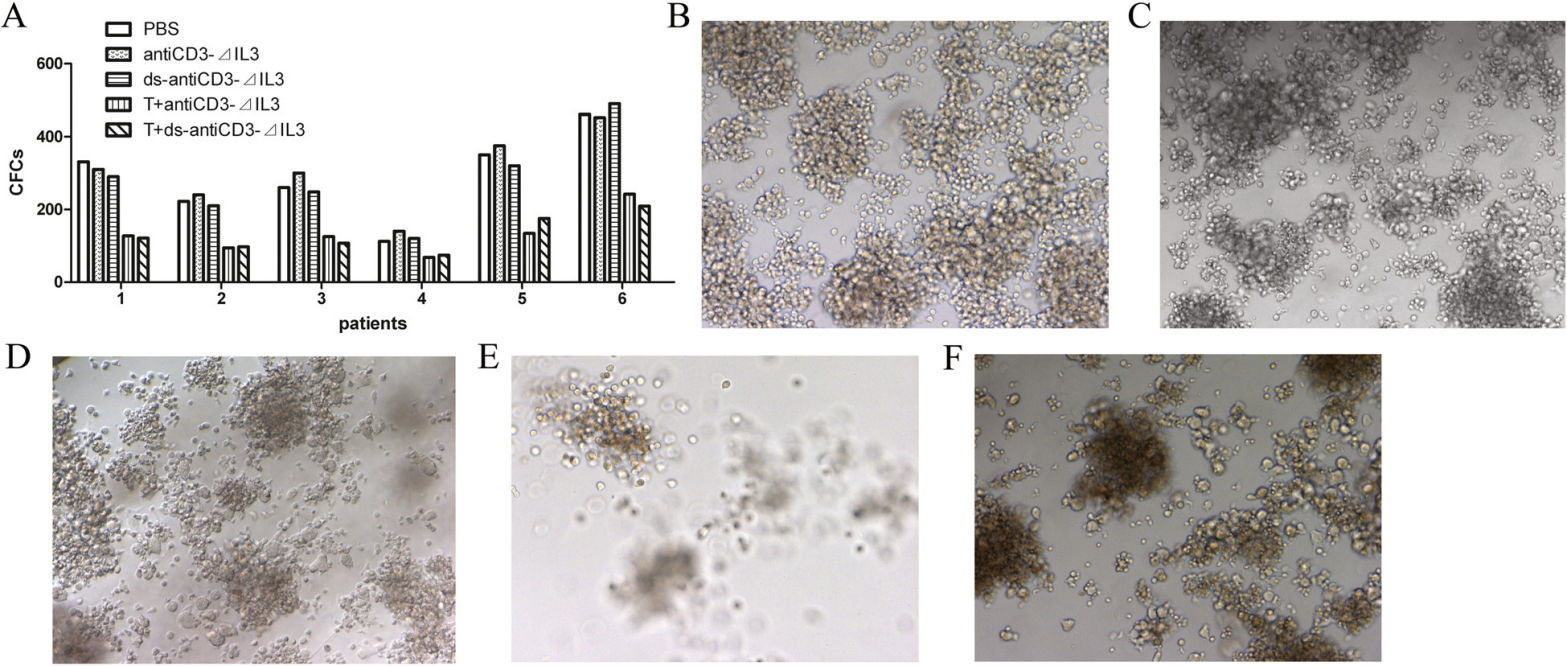
**Cytotoxicity of IL2 pre-activated human T cells mediated by the fusion proteins against AML leukemic progenitors in a methylcellulose colony-forming assay. (A)** AML cells from six patients were incubated in serum-free IMDM for 24 h in the presence or absence of different fusion proteins (500 ng/mL) combined with pre-activated T cells at E/T ratio of 25:1, then plated in AML-CFC assays to evaluate the relative cytotoxicity of T cells mediated by the fusion proteins against these leukemic progenitors. **(B-F)** showed typical colonies of different groups. **(B)** PBS, **(C)** antiCD3Fv-⊿IL3, or **(D)** ds-antiCD3Fv-⊿IL3 alone was used as control group. **(E)** Cytotoxicity of T cells against leukemic progenitors in the presence of antiCD3Fv-⊿IL3. **(F)** Cytotoxicity of T cells against leukemic progenitors in the presence of ds-antiCD3Fv-⊿IL3.

The peripheral blood mononuclear cells from six newly diagnosed AML patients were incubated in serum-free IMDM for 24 h in the presence or absence of different fusion proteins combined with pre-activated T cells. Treated and untreated cells were then plated in AML-colony-forming cell (CFC) assays to evaluate the relative cytotoxicity of T cells mediated by the fusion proteins against these leukemic progenitors. AML-CFCs were detected in control (including PBS-treated or fusion proteins without pre-activated T cells) cultures of all six AML samples, ranging from 112 to 490 AML-CFCs/10^4^ cells (Figure 5A,B,C,D). After 24-h exposure to fusion proteins with pre-activated T cells, the mean percentages of kill of AML-CFCs were 53.3% and 52.8%, respectively, for antiCD3Fv-⊿IL3 and ds-antiCD3Fv-⊿IL3, (range, 33.9% ~ 63.4%) (Figure 5A,E,F). There was no statistical difference between the parental fusion protein and the ds fusion protein (500 ng/mL) in mediating the inhibition of colony formation of AML leukemic progenitors *in vitro* (Figure 5A). These findings indicate that the two fusion proteins can preferentially retarget T cells to AML progenitor cells.

### Inhibition of growth of human KG1a xenografts *in vivo*

**Figure 6:**
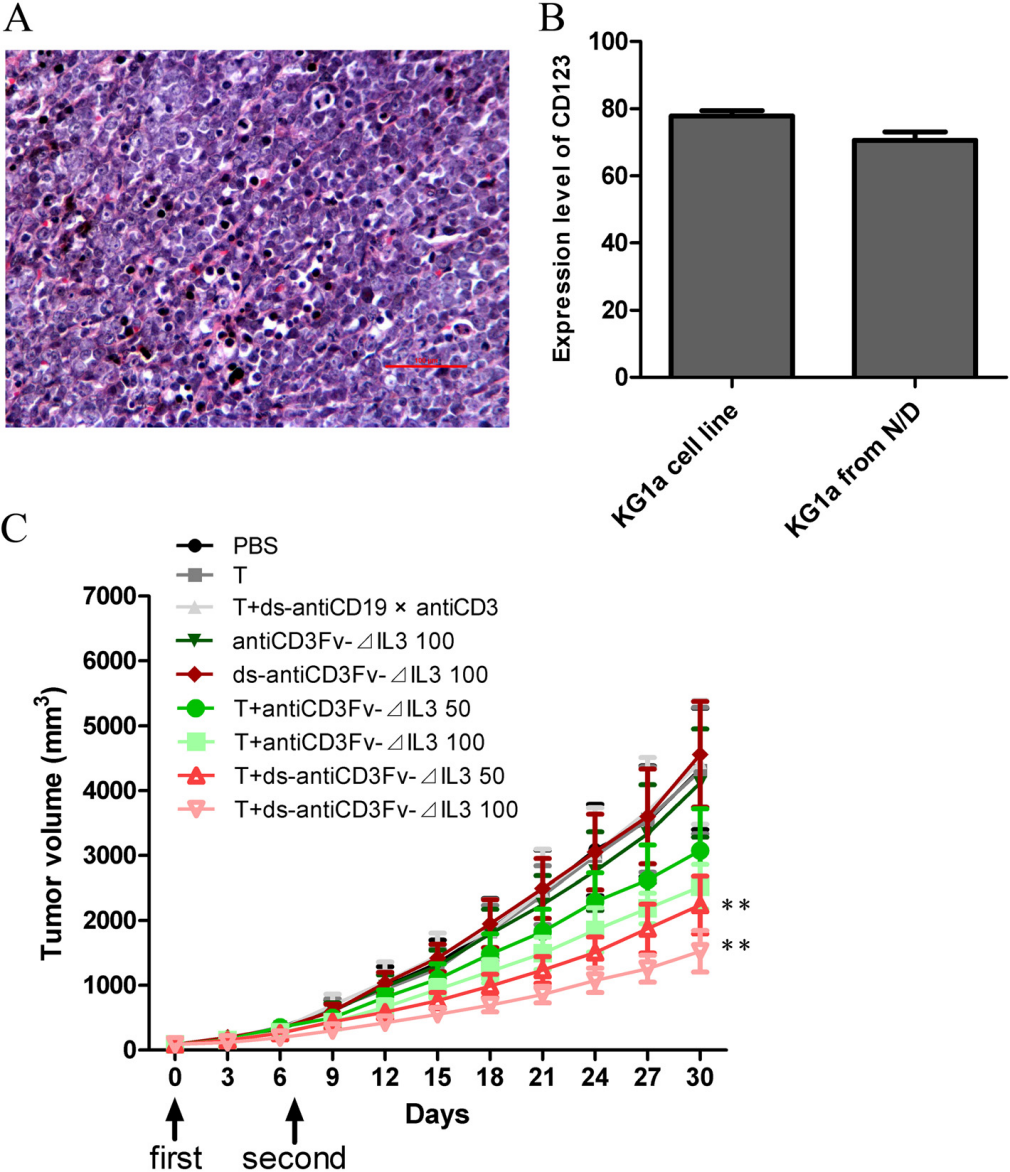
**Antitumor effect of fusion proteins antiCD3Fv-**⊿**IL3 and ds-antiCD3Fv-**⊿**IL3 in KG1a xenografted NOD/SCID mice. (A)** H&E staining showed the KG1a tumor cells were diffused and in intensive distribution, indicating subcutaneous KG1a xenotransplanted model was established successfully. **(B)** The expression level of CD123 on the surface of KG1a xenografts and KG1a cell line were analyzed by FACS. **(C)** A mixture of T cells and fusion proteins antiCD3Fv-⊿IL3 or ds-antiCD3Fv-⊿IL3 in different concentrations (50 and 100 μg/mouse) were injected intravenously 6 days later, when the solid tumors reached 80–100 mm^3^ in size, once per week for 2 weeks. PBS, antiCD3Fv-⊿IL3, ds-antiCD3Fv-⊿IL3, and T cells alone, and T cells combined with antiCD19 × antiCD3 diabody, were used as control groups. The size of the xenografts was measured every 3 days. Error bars indicate SD (*n* = 5). **P < 0.01 vs PBS.

We established a NOD/SCID mouse model bearing the human AML progenitor cell line KG1a to determine whether the fusion proteins also had an antitumor activity *in vivo*. The pathological finding showed KG1a tumor cells were in diffuse and intensive distribution, which indicated the subcutaneous KG1a xenotransplanted model was established successfully (Figure 6A). The expression level of CD123 on the surface of KG1a xenografts (70.620% ± 5.023%) was slightly reduced compared with KG1a cell line (77.875% ± 3.090%) (Figure 6B). Two doses (50 and 100 μg/mouse) of the fusion proteins were chosen to evaluate their ability to inhibit tumor growth in NOD/SCID mice. Animals were sacrificed when the tumors had grown to approximately 4,000 mm^3^ in size. As shown in Figure 6C, compared to the mice in the control group (untreated), the mice treated with different dosages of fusion proteins antiCD3Fv-⊿IL3 or ds-antiCD3Fv-⊿IL3 combined with pre-activated T lymphocytes showed a significant dose-related tumor regression. Furthermore, the ds fusion protein had a better antitumor activity than the parental fusion protein, when equivalent doses of ds fusion protein and the parent fusion protein were compared. The ability of tumor regression still exceeded its parental fusion protein slightly, even with half dose of the ds fusion protein (Figure 6C).

## Discussion

Although current treatment regimens for AML can achieve a complete remission, most patients will ultimately relapse and die from the disease or complications associated with the treatment [2], emphasizing the unmet medical need for novel therapeutics that are more effective and durable. Recent advances in AML-targeting immunotherapies including dendritic cell vaccines, adoptive T cell therapy, and antigen-specific cytotoxic T lymphocytes open the possibilities for eradicating minimal residual diseases which contain chemoresistant LSCs after chemotherapy [26]. To this end, we chose the antigen CD123 out of several cell surface antigens preferentially expressed on AML LSCs compared with normal hematopoietic stem cells, including CD123, CD44, CLL-1, CD47 [7], and T cell immunoglobulin mucin-3 (TIM-3) [27], the rationale of its selection being its overexpression not only by the bulk AML population, but also by LSCs in 75%–89% of AML patients [28-30]. In this study, we aimed to develop a fusion protein antiCD3Fv-⊿IL3 that would direct T cell to specifically target CD123+ AML cells just like bispecific antibodies. Since the heavy chain (V_H_) and the light chain (V_L_) domains of antiCD3Fv are associated non-covalently, they dissociated relatively easily. To enhance the stability of the fusion protein, we employed disulfide covalent bonds to lock the two chains together as we have done before in the antiPgp × antiCD3 diabody and the antiCD19 × antiCD3 diabody, both of which showed a higher antitumor activity in animal models [31,32]. As expected, the disulfide-stabilized antiCD3Fv-⊿IL3 improved the serum stability by the disulfide cross-linking of the CD3 Fv fragments. The results showed that ds-antiCD3Fv-⊿IL3 retained 90% activity even after 3 days post incubation at 37°C in PBS, compared with the parent fusion protein with a t1/2 of 6 h under the same conditions (Figure 3A,B). In addition, the ds-antiCD3Fv-⊿IL3 had the same antigen-binding specificity and cytotoxic activity *in vitro* as the parent fusion protein (Figure 4A,B). Thus, compared with the parent fusion protein, the more stable ds-antiCD3Fv-⊿IL3 may conceivably penetrate deeper into the tumor and stay longer in the tumor, resulting in a greater antitumor activity *in vivo* (Figure 6C).

The expression of multiple cell surface tumor-associated antigens on AML cells has been well characterized [7,26,27]. In this work, we have engineered fusion proteins to redirect T cells to target CD123+ AML cells. The CD123 is highly expressed by primary AML cells, as well as LSCs, while displaying limited expression on hematopoietic stem cells and T cells [7,28,30]. The LSCs are thought to be spared from chemotherapy and capable of reinitiating the disease. Anne S. Rong et al. have discovered CD123 and hMICL in combination with CD34 and CD117 as a universal marker combination for the detection of minimal residual disease (MRD) of AML in pre-clinical and clinical settings [33]. Thus, therapies targeting CD123 aiming at eradicating LSCs should be the successful novel therapeutic strategy, which can be applied after the standard induction chemotherapy to eliminate MRD and reduce recurrence. As expected, therapies targeting CD123 have showed favorable safety profiles in phase I trials (ClinicalTrials.gov ID#NCT00401739 and #NCT00397579). Besides, there are some other AML antigens which may be potential targets. For instance, the epithelial mucin, MUC1, is expressed on primary AML cells and can be recognized by MUC1-specific cytotoxic T lymphocytes [34]. The CD47 is identified as an adverse prognostic factor and is more highly expressed on AML LSC than their normal counterparts [35]. The TIM-3 antigen is another target that can be used to selectively kill AML LSCs, but its expression on a subset of T cells may result in autolysis of T cells [36], making it a non-ideal target for a T-cell-based therapy.

Anticancer immunity involves both the innate and adaptive immune systems, yet it is generally considered that CD8+ cytotoxic T lymphocytes (CTL) are the most potent antitumor effector cells [37]. But in fact, researches have been acutely aware that tumor cells can develop multiple escape mechanisms, such as a physical barrier avoiding infiltration of effector cells and lower expression level of MHC as well as secretion of cytokines that indirectly inhibit T cell proliferation [37]. Therefore, specific reagents for T cell retargeting have been developed to make T cells kill tumors more effectively. Among different approaches, the most potent therapeutics are the use of bispecific antibodies and CARs to redirect T cell specificity toward cell surface tumor-associated antigens. CARs, known as chimeric immune receptors, should be inserted into effector cells, and then, they can redirect effector cell specificity toward cell surface tumor-associated antigens independent of the T cell receptor (TCR)-defined specificity and in a MHC-independent manner. This technology has shown an excellent efficacy in pre-clinical studies as well as clinical trials [38,39]. However, the use of CARs may also be dangerous, such as, the activation of effector cells can be too strong to lead to a cytokine storm and even to death [40]. Therefore, clinical use of CARs is still limited by the complexity of engineering processes and further improvement needs to be done. The efficacy of bispecific antibodies has already been validated in pre-clinical studies and has shown promising results in clinical trials, for example, antiCD19 × antiCD3 BiTE Ab called blinatumomab in phase II trial (ClinicalTrials.gov ID#NCT01207388) has shown an 80% complete molecular response rate and prolonged leukemia-free survival in patients with minimal residual B-lineage acute lymphoblastic leukemia [41]. Besides, there are some other bispecific antibodies under clinical trials, such as antiEpCAM × antiCD3 for epithelial cancers or gastric cancer in phase II/III trial, antiHER-2 × antiCD3 for metastatic or advanced breast cancer in phase II trial [16]. To target CD123, Shu-Ru Kuo et al. have engineered a CD123 × CD3 bispecific scFv immunofusion to target CD123+ LSCs *in vitro*, which contained an antiCD123 scFv fused at the N-terminus of human IgG1 hinge-CH2-CH3, and an antiCD3 scFv fused at C-terminus; the molecular weight of 140 kDa would affect their penetration into tumor tissue [17]. Then, in our work, we successfully constructed a smaller fusion protein antiCD3Fv-⊿IL3 and its disulfide-stabilized antiCD3Fv-⊿IL3, both displaying a potent antileukemic activity, especially for the ds-antiCD3Fv-⊿IL3 *in vivo*. Compared with CD123-specific CAR T cells, this approach is less efficient against AML LSCs but more convenient and controllable in the clinical application. To enhance the curative effect of antiCD3Fv-⊿IL3, it is possible to develop a multi-epitope reagent, because its manner of T cell activation is dependent on T cell receptor functional signals which may be defective in cancer patients.

Moreover, due to the overexpression of CD123 (IL3Rα) on AML leukemic stem cells, researchers have exploited drugs that fuse IL3 to efficient toxin or chemotherapeutics to eradicate human AML stem cells. Among these fusion proteins, the DT388IL3 showed cytotoxicity to LSCs *in vitro* and *in vivo* with minimal damage to normal tissues [10,11]. Currently, phase II clinical trial has been completed. Furthermore, researchers also found that the variant DT388IL3 (△125-133) showed an enhanced binding to the human IL3R and greater cytotoxicity to human AML cell lines and LSCs than the wild-type DT388IL3 [42]. Following the same way, our laboratory constructed and expressed the fusion protein antiCD3Fv-IL3 and discovered the fusion protein was unstable and could easily break in the end of the C-terminal (data not shown), which was also consistent with some combinatorial mutagenesis studies [22-25]. So we engineered the fusion protein antiCD3Fv-⊿IL3 (⊿125-133) and its disulfide-stabilized antiCD3Fv-⊿IL3, which combined the advantages of both the specificity of humoral immunity and the efficiency of cellular immunity compared with fusion proteins DT388IL3.

## Conclusion

Fusion proteins antiCD3Fv-⊿IL3 and disulfide-stabilized antiCD3Fv-⊿IL3, especially the latter one, display the antileukemic activity against CD123-expressing cell lines as well as leukemic progenitors *in vitro* and *in vivo*. They could be the promising candidates for future immunotherapy of AML. In the future, we will explore various methods to enhance T lymphocyte activities against leukemic cells of AML, such as in combination with chemotherapeutics [43] or developing a multi-epitope reagent with ligands of costimulatory molecules.

## Methods and material

### Cell lines

Hybridoma cell lines producing the antiCD3 monoclonal antibody HIT3a (IgG2a, к) were established and maintained at the Institute of Hematology, Chinese Academy of Medical Sciences (Tianjin, China) as described previously [44]. The human acute T cell leukemia cell line Jurkat, human acute myelogenous leukemia progenitor cell line KG1a, human acute monocytic cell line THP1, and human acute promyelocytic leukemia cell line NB4 were all derived from our laboratory. Jurkat, THP1, and NB4 were maintained in RPMI 1640 and KG1a in IMDM media (Gibco, Grand Island, NY) containing 10% fetal bovine serum (Gibco, Grand Island, NY). All the cells were cultured at 37°C with 5% CO_2_.

### Construction of plasmids of the antiCD3Fv-⊿IL3 or disulfide-stabilized antiCD3Fv-⊿IL3

The fragments of antiCD3V_L_ and antiCD3*V_L_ (containing a cysteines at position Ser-100 of CD3V_L_) containing the restriction enzyme site MluI and the G4S linker were obtained by polymerase chain reaction (PCR) from the plasmid pLH-T3a-19a and pLH-T3a*-19a [32], using the primer pairs P1/P2 (Table 1, all primers were synthesized by Invitrogen Life Technologies, USA), respectively. The antiCD3V_H_ and antiCD3*V_H_ (containing a cysteines at position Gly-44 of CD3V_H_) segments containing the restriction enzyme site MluI and the G4S linker were generated similarly by PCR from the plasmid pLH-19a-T3a and pLH-19a-T3a* [32], respectively, using the primer pairs P5/P6. The fragment of ⊿IL3 (the end of eight amino acids were deleted) was obtained by PCR from cDNA of human peripheral blood mononuclear cells, using the primer pairs P3/P4 (including the restriction enzyme site NheI and the G4S linker) or P3/P7 (both containing the G4S linker). The His tag and the restriction enzyme site SphI were introduced into the PCR products by re-amplification with the primer pairs P3/P8. Overlap PCR was used to generate the fragments of antiCD3V_L_-G4S-⊿IL3 or antiCD3*V_L_-G4S-⊿IL3 and antiCD3V_H_-G4S-⊿IL3-His or antiCD3*V_H_-G4S-⊿IL3-His through the complementary sequences of the G4S linker. The resulted fusion products were cleaved with the restriction enzymes MluI/NheI and MluI/SphI and subsequently cloned into the pLH-T3a-19a and pLH-19a-T3a vectors to generate plasmids pLH-CD3V_L_ or CD3*V_L_-⊿IL3 and pLH-CD3V_H_ or CD3*V_H_-⊿IL3-His. The latter vector has contained the restriction enzymes site NheI and the *E. coli* heat-stable enterotoxin II (*stII*) signal at the 5′ end of antiCD3V_H_ or antiCD3*V_H_-⊿IL3-His. The abovementioned vectors were cleaved with the restriction enzymes MluI/NheI and NheI/SphI, respectively, to generate two fragments of antiCD3V_L_ or antiCD3*V_L_-⊿IL3 and stII-antiCD3V_H_ or antiCD3*V_H_-⊿IL3-His and then subcloned into the MluI/SphI sites of the expression vector pCANTAB5E (GE healthcare, Little Chalfont, Buckinghamshire, UK).

**Table 1:** **Primers used for construction of fusion proteins expression vectors**

**Primer names**	**Primer sequence (5′ → 3′)**
P_1_	CTTCGAGCTAGCTACCCGGGGATCCTCTAGAG
P_2_	CACCCTCCGCCACCCCG
P_3_	GGTGGCGGAGGGTCGGCTCCCATGACCCAGACAAC
P_4_	GCGCGCTAGCTCACTGTTGAGCCTGCGCATTCTC
P_5_	GCGCACGCGTACGCTCAGGTGCAGCTGCAGCAGT
P_6_	CCCTCCGCCACCTGAGGAGACGGTGACCGTG
P_7_	TGATCCGCCTCCACCAAAGATCGCGAGGCTCAAAGT
P_8_	GCGCGCATGCTCAATGATGGTGATGGTGATGTGATCCGCCTCCACC

### The expression and purification of fusion proteins

The soluble fusion protein was extracted from *E. coli* strain 16C9 containing the expression plasmids and purified by methods described previously [31]. The purified fusion proteins were subjected to electrophoresis in 12% polyacrylamide gels and stained with Coomassie brilliant blue to confirm their purity, respectively. In addition, an identical gel was transferred onto a PDVF membrane and a Western blot analysis was performed using a mouse anti-His-tag antibody IgG (Tiangen Biotech, Beijing, China) and goat antimouse antibody IgG conjugated with horseradish peroxidase (Union Stem Cell & Gene engineering, Tianjin, China). Purified fusion proteins were kept under a sterile condition and stored at −80°C.

### Flow cytometric analysis

The CD123-positive cell line KG1a and the CD3-positive cell line Jurkat were employed for analysis of binding activity of the purified proteins by flow cytometry (FACS LSRII, Becton Dickinson Bioscience, San Jose, CA). Briefly, 1 × 10^6^ cells in 100-μL phosphate-buffered saline (PBS) were incubated with various fusion proteins (20 μg/mL) for 1 h at 4°C. After being washed three times with cold PBS, the cells were incubated with 100-μL anti-His antibody IgG at 10 μg/mL for 1 h, followed by incubation with 20 μL of FITC-labeled rabbit antimouse antibody IgG (Institute of Hematology, Chinese Academy of Medical Sciences, Tianjin, China) for an additional 30 min. The stained cells were washed and analyzed by flow cytometry. In a competitive binding assay, 1 × 10^6^ KG1a cells in 100-μL PBS were first incubated with 20 μg/mL of various fusion proteins for 1 h at 4°C, then incubated with parent McAb IgG (CD123 McAb for KG1a and HIT3a for Jurkat cells, respectively) for 1 h at 4°C. After being washed three times with cold PBS, the cells were incubated with an FITC-conjugated rabbit antimouse IgG for 30 min. The stained cells were then analyzed by flow cytometry.

### Stability assays *in vitro*

Fusion proteins were incubated at a concentration of 20 μg/mL in PBS containing 0.2% (*w*/*v*) HSA at 37°C for a variety of time periods. The remaining binding activity of the fusion proteins incubated with KG1a or Jurkat cells were detected using flow cytometry.

### Isolation of PBMC, sorting, and stimulation of T lymphocytes

With informed consent obtained, human peripheral blood mononuclear cells (PBMCs) were isolated from peripheral blood taken from healthy volunteers or AML patients using Ficoll-Hypaque density-gradient centrifugation (Institute of Hematology, Chinese Academy of Medical Sciences, Tianjin, China). The clinical processes were approved by the Ethics Committees of the Hospital of Blood Disease, Peking Union Medical College. The mononuclear cell layer was removed, washed twice, and cell number counted, then the cell suspension was depleted of monocytes by adherence to plastic flasks for 2 h. Non-adherent cells were used for T cell isolation by fluorescence-activated cell sorter (FACS). PBMCs (1 × 10^8^) in 500-μL PBS were incubated with 15 μL of allophycocyanin (APC)-conjugated antihuman CD3 mAbs (clone UCHT1,BD Pharmingen, San Diego, CA) at 4°C for 60 min and then washed twice with PBS. T cells sorted out by FACS (AriaIII, Becton Dickinson Biosciences) were cultured in a complete RPMI 1640 medium containing 10% FCS and 50 IU/mL of IL2 for 48 h to activate T lymphocytes.

### Cytotoxicity assay *in vitro*

The efficacy of fusion proteins in mediating T cell cytotoxicity was evaluated by the calcein-release assay [45]. The CD123-positive cell line KG1a and the CD123-negative cell line THP1 and NB4 were prepared as target cells. Equal concentrations of antiCD3 × antiCD19 diabody were established as control. The experimental and calculating methods were proceeded as described previously [31].

### Methylcellulose colony-forming assay

To evaluate inhibition of colony formation, the mononuclear cells from peripheral blood of an AML patient were cultured in serum-free IMDM for 24 h in the presence or absence of different fusion proteins (500 ng/mL) combined with pre-activated T cells at the effector-target cell ratio of 3:1. Then, cells were plated in 96-well plates at 10,000 cells/25 μL or 40,000 cells/25 μL in Methocult H4434. And the numbers of colonies were scored after 15–20 days of culture. All the treatments were performed in triplicate.

### Growth inhibition of human KG1a xenografts *in vivo*

All animal procedures were approved by Ethics Committee on the use and care of animals, Chinese Academy of Medical Science. Female 6-week-old NOD/SCID mice (Cancer Institute, Chinese Academy of Medical Sciences, Beijing, China) were inoculated subcutaneously with 2 × 10^7^ KG1a cells into armpits, 1 day after the application of total body irradiation (300 cGy). Treatment was initiated at 6 days post-tumor inoculation, when the solid tumors reached 80–100 mm^3^ in size. Each group consisted of seven mice at least. Mice were treated with two different doses (50, 100 μg/mouse) of the two fusion proteins, combined with pre-activated T cells (5 × 10^6^ cells/mouse) in PBS via the tail vein, every 7 days for 2 weeks. Mice received intravenous injections of various fusion proteins (100 μg/mouse) alone, pre-activated T cells (5 × 10^6^ cells/mouse), a combination of antiCD3 × antiCD19 (100 μg/mouse) diabody and pre-activated T cells (5 × 10^6^ cells/mouse), or PBS as control. Growing tumors were measured using a vernier caliper in two perpendicular dimensions every 3 days, and the tumor volumes were calculated using the following formula: *V* = (*L* × *W*^2^)/2, where *L* represents the longest axis of the tumors (in mm) and *W* represents the axis perpendicular to *L* (in mm). Mice were sacrificed by cervical dislocation under anesthesia when the tumors reached 4,000 mm^3^ in size. The mean tumor volume of the treatment groups and control groups were evaluated by Student’s *t*-test with SPSS18.0 software (SPSS, Cary, NC).

## Additional files

Additional file 1: Movie 1. The process of cytotoxicity of IL2 pre-activated of human T cells to KG1a cells mediated by the fusion protein antiCD3Fv-⊿IL3 was observed by Nikon Ti-e cell-imaging system. Cells (5 × 10^3^ KG1a per well) were plated in a 96-well plate, followed by added IL2 pre-activated human T cells at effector-target cell ratio of 25:1 in the presence of antiCD3Fv-⊿IL3 (500 ng/mL). After a transient laid aside in the incubator, the plate was observed by Nikon Ti-e cell-imaging system. Several fields were chosen and a picture was taken for each every 1 h. The actual speed of the movie plays at roughly 500 ms/frame. In the movie, the larger cells were KG1a cells and the smaller cells were T cells. The process of recruitment of T cell to tumor cells and the lysis of tumor cells was observed. Additional file 2: Movie 2. The process of cytotoxicity of IL2 pre-activated of human T cells to KG1a cells mediated by the fusion protein ds-antiCD3Fv-⊿IL3 was observed by Nikon Ti-e cell-imaging system. The same as antiCD3Fv-⊿IL3, we can observe the process of recruitment of T cell to tumor cells and the lysis of tumor cells in this movie. The larger cells were KG1a cells and the smaller cells were T cells. Additional file 3: Figure S1. The typical screenshots of movie 1 (A, B, C, D) displayed the cytotoxicity of T cells mediated by the fusion protein antiCD3Fv-⊿IL3 in one field at different time points. The blue areas showed the typical process of cytotoxicity. The larger cells were KG1a cells and the smaller cells were T cells. The fusion protein antiCD3Fv-⊿IL3 appeared to be potent in retargeting T cell lysis of the KG1a cells. Additional file 4: Figure S2. The typical screenshots of movie 2 (A, B, C, D) displayed the cytotoxicity of T cells mediated by the fusion protein ds-antiCD3Fv-⊿IL3 in one field at different time points. The blue areas showed the typical process of cytotoxicity. The same as antiCD3Fv-⊿IL3, ds-antiCD3Fv-⊿IL3 also appeared to be potent in retargeting T cell lysis of the KG1a cells.

## Abbreviations

AML: Acute myeloid leukemia
CAR: Chimeric antigen receptor
CFCs: Colony-forming cells
CTL: Cytotoxic T lymphocyte
ds: Disulfide-stabilized
IL3: Interleukin-3
LSCs: Leukemic stem cells
MRD: Minimal residual disease
MHC: Major histocompatibility complex
NOD/SCID: Non-obese diabetic/severe combined immunodeficient
PBMC: Peripheral blood mononuclear cells
PCR: Polymerase chain reaction
scFv: Single-chain Fv
