# Supplementary figures and images for: AntiCD3Fv fused to human interleukin-3 deletion variant redirected T cells against human acute myeloid leukemic stem cells
Source: J Hematol Oncol. 2015 Feb 28;8:18. doi: 10.1186/s13045-015-0109-5 (PMC4389834; doi:10.1186/s13045-015-0109-5)

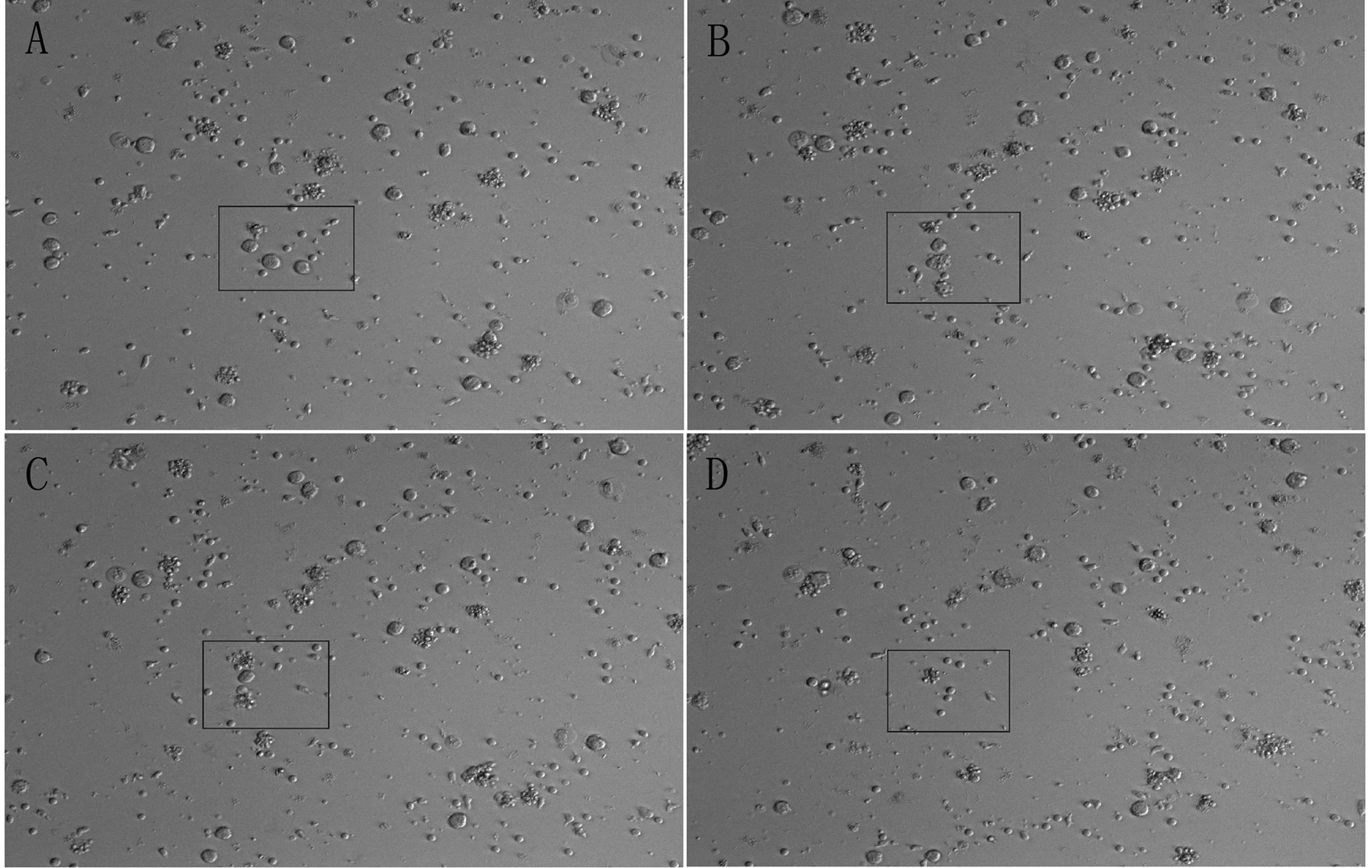

Supplement: Additional file 3: Figure S1. — The typical screenshots of movie 1 (A, B, C, D) displayed the cytotoxicity of T cells mediated by the fusion protein antiCD3Fv-⊿IL3 in one field at different time points. The blue areas showed the typical process of cytotoxicity. The larger cells were KG1a cells and the smaller cells were T cells. The fusion protein antiCD3Fv-⊿IL3 appeared to be potent in retargeting T cell lysis of the KG1a cells. [file 13045_2015_109_MOESM3_ESM.jpeg]

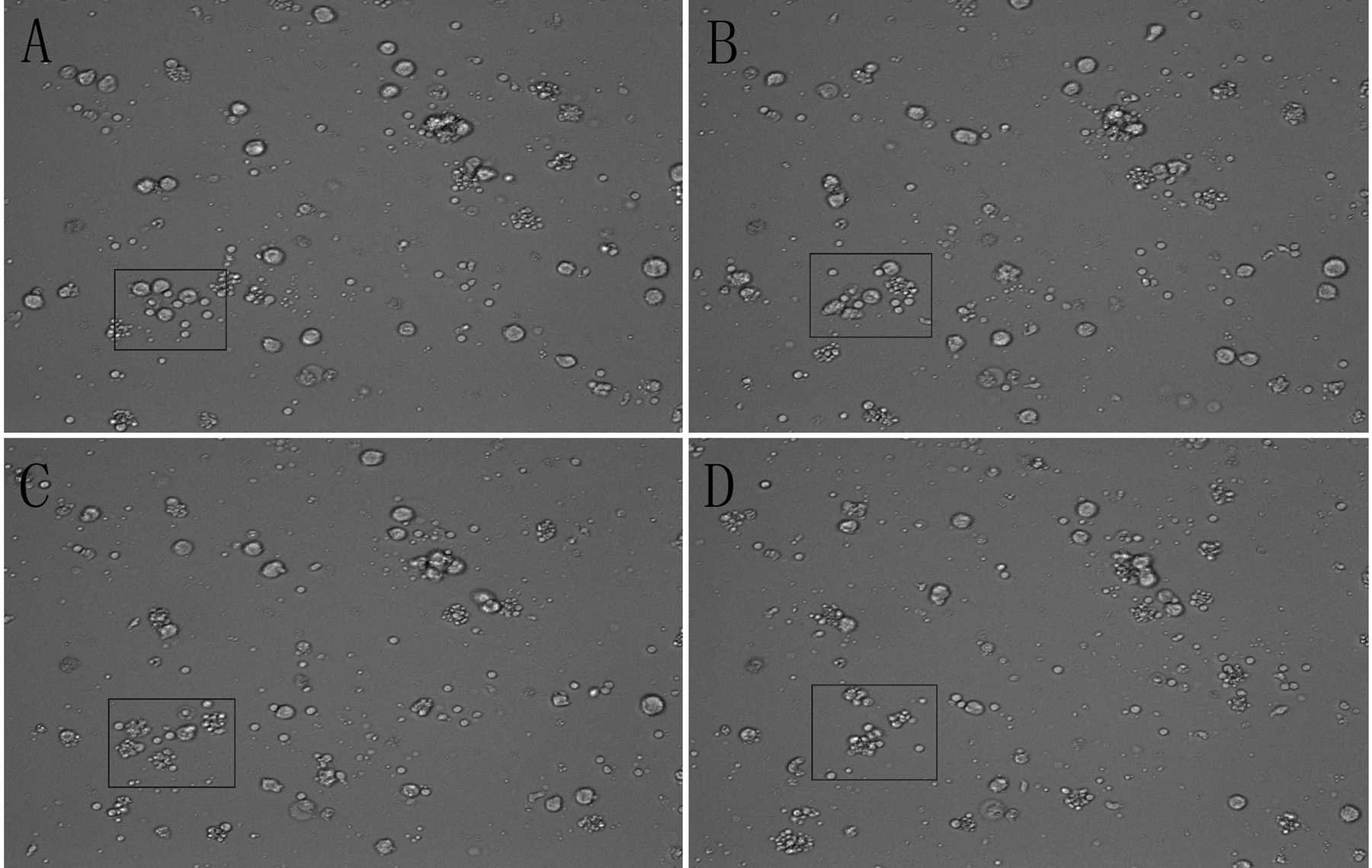

Supplement: Additional file 4: Figure S2. — The typical screenshots of movie 2 (A, B, C, D) displayed the cytotoxicity of T cells mediated by the fusion protein ds-antiCD3Fv-⊿IL3 in one field at different time points. The blue areas showed the typical process of cytotoxicity. The same as antiCD3Fv-⊿IL3, ds-antiCD3Fv-⊿IL3 also appeared to be potent in retargeting T cell lysis of the KG1a cells. [file 13045_2015_109_MOESM4_ESM.jpeg]
